# Cardioprotective effects of sinomenine in myocardial ischemia/reperfusion injury in a rat model

**DOI:** 10.1016/j.jsps.2022.04.005

**Published:** 2022-04-21

**Authors:** Changhong Lu, Xiao Guo, Xianghui He, Yu Chang, Fa Zheng, Chenji Xu, Shuwen Zhang, Yaqun Zhou, Junfang Li

**Affiliations:** a Department of Heart Center, Qingdao Fuwai Cardiovascular Hospital, No.201 Nanjing Road, Qingdao City, Shandong Province 266034, China; b Department of Emergency, Qingdao Fuwai Cardiovascular Hospital, No.201 Nanjing Road, Qingdao City, Shandong Province 266034, China; c Department of Cardiac Ultrasound, The Affiliated Hospital of Qingdao University, No. 16 Jiangsu Road, Qingdao City, Shandong Province 266000, China

**Keywords:** Sinomenine, Ischemia reperfusion, Arrhythmias, Antioxidant, Plasmin system, I/R, Ischemia reperfusion, SM, Sinomenine, NC, Normal control, VEB, Ventricular ectopic beat, CK-MB, Creatine kinase MB, CK, Creatine kinase, Tnl, Troponin I, TF, Tissue factor, TXB2, Thromboxane B2, PAI-1, Plasminogen activator inhibitor 1, Fbg, Plasma fibrinogen, ATP, Adenosine triphosphate, MII, Myocardial ischemic injury, IHD, Ischemic heart disease, LAD, Left anterior descending coronary artery, K-H, Krebs-Henseleit, PVC, Premature ventricular contraction, MDA, Malonaldehyde, GPx, Glutathione peroxidase, CAT, Catalase, SOD, Superoxide dismutase, AST, Aspartate aminotransferase, LDH, Lactate dehydrogenase, IL-1β, Interleukin-1β, MCP-1, Monocyte chemoattractant protein-1, hs-CRP, C-reactive protein, IL-6, Interleukin-6, TNF-α, Tumor necrosis factor-α, SD, Standard deviation, WHO, World Health Organization, PCI, Percutaneous coronary intervention

## Abstract

**Background:**

Ischemia reperfusion (I/R) play an imperative role in the expansion of cardiovascular disease. Sinomenine (SM) has been exhibited to possess antioxidant, anticancer, anti-inflammatory, antiviral and anticarcinogenic properties. The aim of the study was scrutinized the cardioprotective effect of SM against I/R injury in rat.

**Methods:**

Rat were randomly divided into normal control (NC), I/R control and I/R + SM (5, 10 and 20 mg/kg), respectively. Ventricular arrhythmias, body weight and heart weight were estimated. Antioxidant, inflammatory cytokines, inflammatory mediators and plasmin system indicator were accessed.

**Results:**

Pre-treated SM group rats exhibited the reduction in the duration and incidence of ventricular fibrillation, ventricular ectopic beat (VEB) and ventricular tachycardia along with suppression of arrhythmia score during the ischemia (30 and 120 min). SM treated rats significantly (P < 0.001) altered the level of antioxidant parameters. SM treatment significantly (P < 0.001) repressed the level of creatine kinase MB (CK-MB), creatine kinase (CK) and troponin I (Tnl). SM treated rats significantly (P < 0.001) repressed the tissue factor (TF), thromboxane B2 (TXB2), plasminogen activator inhibitor 1 (PAI-1) and plasma fibrinogen (Fbg) and inflammatory cytokines and inflammatory mediators.

**Conclusion:**

Our result clearly indicated that SM plays anti-arrhythmia effect in I/R injury in the rats via alteration of oxidative stress and inflammatory reaction.

## 1 Introduction

As per the World Health Organization (WHO) reports, almost 2.3 million deaths are related to ischemic heart disease every year (Badalzadeh et al., 2014, Tang et al., 2020). Ischemia reperfusion (I/R) takes part in mortality, tissue injury and morbidity in various types of cardiovascular diseases especially myocardial infarction (Badalzadeh et al. 2014). Tissue injury occurs as a result of the initial ischemia insult, which is dictated by the length and magnitude of the blood supply disruption, and the subsequent injury caused by reperfusion (Granier et al., 2013, Tse et al., 2016a). The accumulation of lactate and anaerobic metabolism causes a decrease in intracellular adenosine triphosphate (ATP) and pH during prolonged ischemia (Tse et al. 2016b). Furthermore, the ATPase dependent ion transport mechanisms become impaired, contributing to enhanced calcium overload (intra-mitochondrial calcium and intracellular level, cell rupture and swelling, and apoptotic, cell death via necrotic, necrotic, autophagic and necroptotic mechanisms (Najafi et al., 2018, Williams et al., 2020). During reperfusion, the restoration of oxygen molecules results in the creation of reactive oxygen species (ROS) (Najafi et al. 2018). Inflammatory cytokines also promote neutrophil infiltration into ischemic tissue, speeding up the I/R damage (Wu et al. 2017). Arrhythmias, transitory mechanical impairment of the heart, microvascular damage and “noreflow” phenomena, as well as an inflammatory reaction, are all caused by I/R injury (Lu et al. 2020). During the reperfusion phase of I/R damage, autophagy, apoptosis, and necrosis all cause cell death (Liu et al. 2019). Recent years have seen significant enhancements in the protective strategies to suppress all features of post ischemic injury in cardiovascular diseases (Gatzke et al., 2018, Liu et al., 2019). Due to the scarcity of therapy options, a safer and more effective technique for developing cardiovascular drugs is urgently needed (Badalzadeh et al., 2014, Geldi et al., 2018).

Myocardial ischemic injury (MII) is causing the greatest number of deaths and disabilities in the world (Yang et al. 2018). I/R damage causes myocardial injury, which is a pathological state of coronary artery disease (Badalzadeh et al. 2014). The most common alterations associated with ischemic heart disease (IHD) include metabolite deposition, decreased intracellular [K+] and pH, irreversible cellular injury, Ca2+ overload and increased oxidative stress by increasing the generation of ROS (Badalzadeh et al. 2014). During the I/R injury, an imbalance between the endogenous antioxidant and ROS occurred (Chang et al., 2002, Vilskersts et al., 2009). During the disease, start the production of ROS due to the continuous generation of free radicals (Badalzadeh et al. 2014). Therefore, the therapy available for ischemia such as reperfusion and it has contrary aspects that can suppress the protective effect of myocardial reperfusion such as myocardial stunning, remodelling of left ventricular extracellular matrix, microvascular impairment, progressive cell death, ventricular arrhythmias and finally cause death (Badalzadeh et al., 2014, Li et al., 2019, Tang et al., 2020).

Ventricular arrhythmias are split into three distinct phases during ischemia: phase 1a arrhythmias occur during the first 10 min, phase 1b arrhythmias occur between 15 and 60 min (beginning of ischemia), and phase 2 arrhythmias occur after 90 min. According to research, myocardial arrhythmias are the most common complication of I/R damage (Lu et al. 2020). Arrhythmias enhance the ROS production, which further start the production of H + gradient, endow to an influx of Na + and increase the [Ca2 + ]i via 2Na^+^/Ca^2+^ exchanger which resultant start the accumulation of [Ca2 + ]I and start the diminution of ATP (Badalzadeh et al., 2014, Han et al., 2019, Li et al., 2019). Due to increase [Ca2 + ]i is consider as the potential target for reperfusion arrhythmogenesis. Clinically, myocardial arrhythmias is the serious problem of I/R injury and 80% of patients suffering from the acute myocardial infarction. Additionally, free radicals and inflammatory reaction have been compromised in the pathophysiology of cardiac cell death, electrophysiological dysregulation and post ischemic contractile impairment (Lu et al. 2020). According to previous study, the inflammatory response plays a crucial role in the I/R damage (Badalzadeh et al., 2014, Han et al., 2019). Actually, the incidence of arrhythmias in myocardial reperfusion might have a direct effect on the enhanced inflammatory reaction and production of ROS during myocardial I/R (Han et al., 2019, Liu et al., 2019). During the reperfusion injury, increase the inflammatory reaction which further activates the NF-κB and results in an increase in chemokine genes and inflammatory cytokines and boost the myocardial injury (Badalzadeh et al., 2014, Qiao et al., 2019).

Sinomenine (IUPAC name (7,8-didehydro-4-hydroxy3,7-dimethoxy-17-methyl-9a, 13a, 14a-morphinan-6-one) isolated from the *Sinomenium acutum* (a Chinese herb) (Li et al., 2021, Zhou et al., 2020). The sinomenine is very popular herb among the Chinese doctors to treat the various inflammatory disease such as rheumatic (Zhou et al., 2020, Lin et al., 2008). Some pharmacological investigation showed that it has remarkable anti-inflammatory, antiarthritic and analgesic effect (Geng et al., 2021, Li et al., 2021). Last few decades, sinomenine widely used for the treatment of chronic glomerulonephritis, allograft rejection, autoimmune nephritis condition and mesangial proliferative nephritis (Zhou et al., 2020, Zhang et al., 2012, Lin et al., 2008, Li et al., 2021). Recent investigation showed that sinomenine suppress the synovial and lymphocyte fibroblast proliferation, macrophage infiltration and the production of inflammatory cytokines (Geng et al., 2021, Yang et al., 2017, Li et al., 2021). As my best knowledge, the myocardial protective effect of sinomenine against the I/R induced ventricular arrhythmias not explored. In this experimental study, we try to explore the cardio-protective effect of sinomenine against the I/R injury rat model and explore the underling mechanism.

## 2 Materials and methods

### 2.1 Drugs and chemical compounds

Sinomenine was purchased from the Sigma Aldrich (St. Louis, USA).

### 2.2 Experimental animal

Wistar rats (250 ± 50 g; sex both) were used in this protocol. The rats were received the standard controlled diet (Table 1) and water *ad libitum*. The rats were kept in the controlled laboratory condition (temperature 22 ± 5 °C; 65% relative humidity; 12/12 h light/dark cycle). The experimental study was carried out according to the International standard animal protocol (QFCH2021A0901).

**Table 1:** List of experimental group.

**S. No**	**Group**	**Symptoms**
**1**	Normal	1% Acacia
**2**	I/R	1% Acacia
**3**	I/R + SM	5 mg/kg
**4**	I/R + SM	10 mg/kg
**5**	I/R + SM	20 mg/kg

### 2.3 Myocardial ischemia/reperfusion

The rats were kept under intermittent positive pressure ventilation with room air, after the intratracheal cannula was placed. Myocardial ischemia was caused by externalising the heart using a left thoracic incision and a slipknot (5–0 silk) around the left anterior descending coronary artery (LAD) (Yang et al. 2018).

### 2.4 Experimental protocol

The rats were divided into following groups presented in Table 1. The rats were acclimated for 7 days before the experimental protocol. All the experimental and surgical protocols were adapted as per the international animal guidelines.

### 2.5 Langendorff heart perfusion

All the experimental rats were heparinized (500 IU) and after that anesthetized using the 60 mg/kg ketamine and 10 mg/kg xylazine mixture, and then hearts were successfully isolated from all rats and immediately mounted on the Langendorff apparatus. The heart tissues were perfused using the Krebs-Henseleit (K-H) solution. Also, a mixture of CO2 (5%) and O2 (95%) was bubbled via perfusion for maintaining the pH 7.4. a thermostatically controlled water circulator was used for maintained the temperature (37 °C) and perfusate (Badalzadeh et al. 2014).

### 2.6 Ventricular arrhythmias

Lambeth conventions was used for classified the ventricular arrhythmias. Ventricular extopic beat (VEB) was classified as the identifiable premature QRS complexes. Ventricular tachycardia (VT) was considered as the incidence of 4 or more consecutive VEBs at a rate faster that the resting sinus rate. Another arrhythmias parameter like ventricular fibrillation (VF) was low voltage and unidentifiable QRS complexes. The VEBs pattern such as couplet, salvos and bigeminy were analysed.

### 2.7 Arrhythmia score

The previous reported method was used for the estimation of arrhythmias score with minor modification. The arrhythmias were scrutinized using the Lambeth Conventions and arrhythmia severity was showed on the basis of Walker and Curtis criteria. 5 grade evaluation system was used for arrhythmias scoring presented in Table 2 (Yang et al. 2018).

**Table 2:** showed the arrhythmia score.

**S. No**	**Score**	**Symptoms**
**1**	0–1	No arrhythmia
**2**	2	VEB
**3**	3	VB or VS
**4**	4	VT
**5**	5	VF

### 2.8 Oxidative stress parameter

The standard available kits were used for the determination of antioxidant enzymes includes malonaldehyde (MDA), glutathione peroxidase (GPx), catalase (CAT) and superoxide dismutase (SOD) using the manufacture protocol (Nanjing Jiancheng Biological Product, Nanjing, China).

### 2.9 Hepatic and heart parameters

Hepatic parameter such as aspartate aminotransferase (AST) and heart parameters includes Tnl, CK, LDH and CK-MB were analyzed using the available kits following the given instruction (Beyotime Biotechnology, Shanghai, China).

### 2.10 Fibrinolytic enzyme and coagulation system indicators

Coagulation system indicators and fibrinolytic enzyme such as TXB2, TF, PAI-1 and Fbg were estimated using the ELISA kits following the manufacture instruction (Beijing Expand biotech Ltd. Beijing, China).

### 2.11 Inflammatory parameters

Interleukin-1β (IL-1β), monocyte chemoattractant protein-1 (MCP-1), interleukin-6 (IL-6) and tumor necrosis factor-α (TNF-α) were determined using the manufacture instruction (Tsz Biosciences, Greater Boston, USA).

### 2.12 Statistical analysis

The data was presented as mean standard deviation (SD) and analysed using one way ANOVA, followed by the Tukey test in GraphPad Prism 8.0 software. P < 0.05 was consider as significant.

## 3 Result

### 3.1 Ventricular arrhythmias

**Fig. 1:**
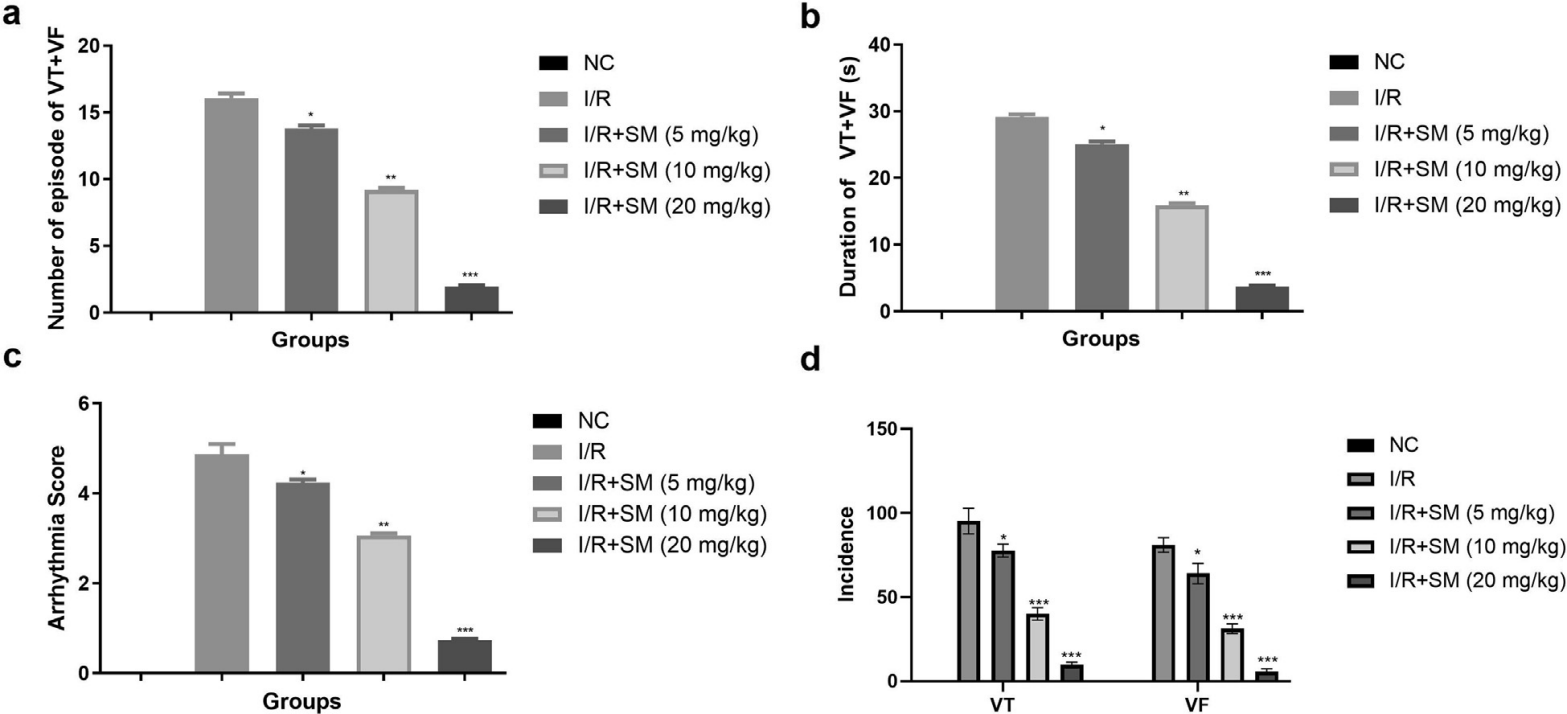
Exhibited the parameters of VT + VF during 30 min ischemia. **a:** number of episodes of VT + VF, **b:** duration of VT + VF (s), **c:** arrhythmia score and **d:** incidence. Data were presented as mean ± SEM. Tested group rats compared I/R where *P < 0.05, **P < 0.01 and ***P < 0.001. Where VT = Ventricular tachycardia, VF = Ventricular fibrillation, NC = Normal Control, I/R = Ischemia reperfusion, SM = Sinomenine.

**Fig. 2:**
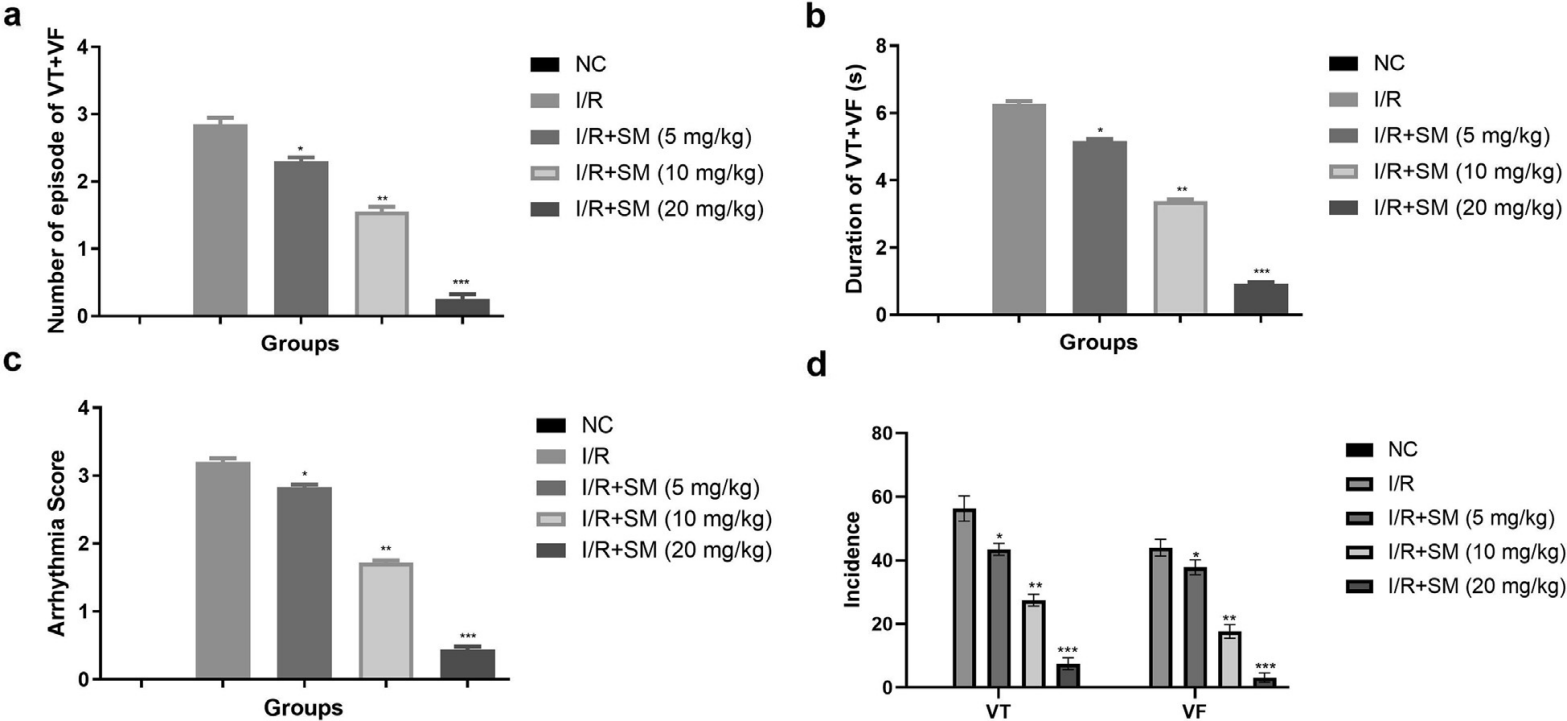
Exhibited the parameters of VT + VF during 120 min ischemia. **a:** number of episodes of VT + VF, **b:** duration of VT + VF (s), **c:** arrhythmia score and **d:** incidence. Data were presented as mean ± SEM. Tested group rats compared I/R where *P < 0.05, **P < 0.01 and ***P < 0.001. Where VT = Ventricular tachycardia, VF = Ventricular fibrillation, NC = Normal Control, I/R = Ischemia reperfusion, SM = Sinomenine.

The effect of sinomenine on the number of episodes of VT + VF in 30 min of ischemia was shown in Fig. 1. After 30 min of ischemia, normal rats showed no signs of VT + VF episodes. VT + VF episodes, duration and incidence (VF and VT) were observed higher in the I/R group rats after 30 min of ischemia. I/R-induced rats had a higher arrythmia score, while sinomenine-treated rats had a lower the VT + VF episode, duration, incidence (VF and VT) and arrythmia score. In 30 min of ischemia, Sinomenine (20 mg/kg) treated group significantly (P < 0.001) reduced the VT + VF episodes, duration, and incidence (VF and VT) Fig. 2.

A similar momentum was observed, after the 120 min ischemia. In 120 min of ischemia, the I/R group exhibited the boosted VT + VF episode, duration and VT, VF incidence along with the enhanced arrythmia score. In 120 min of ischemia, Sinomenine therapy reduced the episode, occurrence, and duration of VT + VF. I/R induced rats exhibited the enhanced arrythmia score in 120 min ischemia and sinomenine treated rats suppressed the arrythmia score (Fig. 2).

### 3.2 Ventricular ectopic beat

**Fig. 3:**
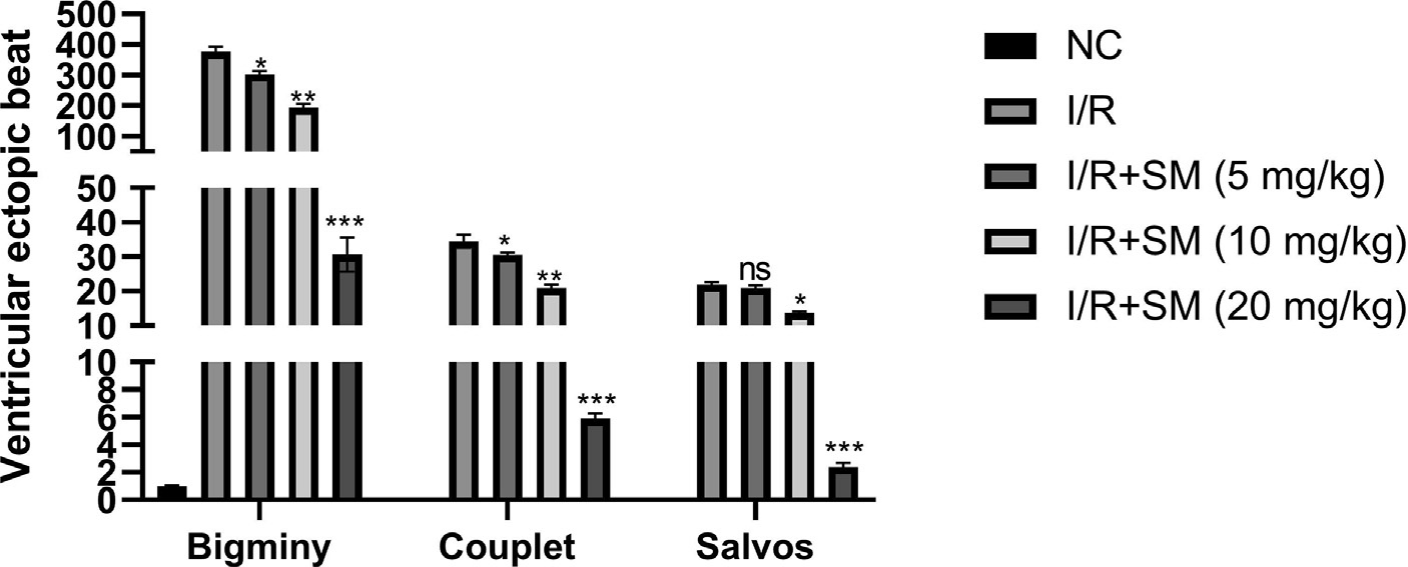
Exhibited the ventricular ecotopic beat. Data were presented as mean ± SEM. Tested group rats compared I/R where *P < 0.05, **P < 0.01 and ***P < 0.001. Where I/R = Ischemia reperfusion, SM = Sinomenine, NC = Normal Control, NS = Non-significant.

I/R induced rats demonstrated the reduction of bigminy, couplet and salvos as compared to normal group rats. I/R induced rats treated with the sinomenine significantly (P < 0.001) suppressed the bigminy, couplet and salvos (Fig. 3). Sinomenine (20 mg/kg) group rats exhibited the maximum reduction in the bigminy, couplet and salvos as compared to the sinomenine (5 and 10 mg/kg) group.

### 3.3 Myocardial infarct area

**Fig. 4:**
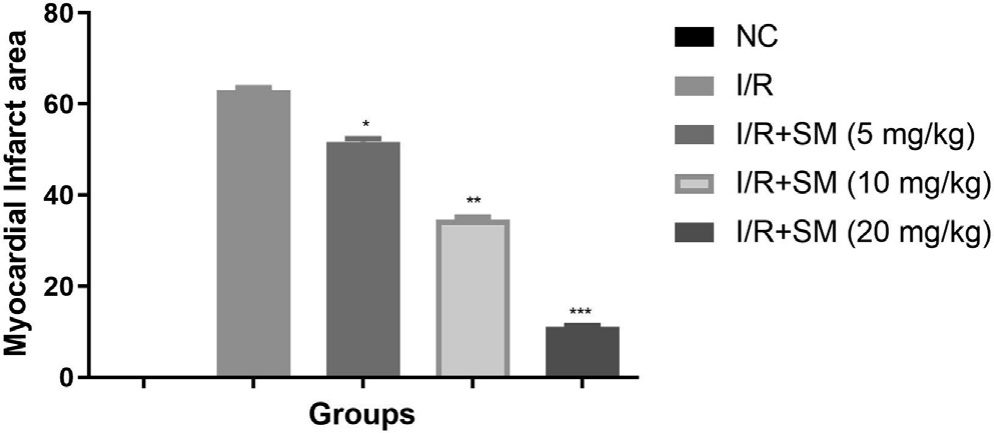
Exhibited the myocardial infract area. Data were presented as mean ± SEM. Tested group rats compared I/R where *P < 0.05, **P < 0.01 and ***P < 0.001. Where I/R = Ischemia reperfusion, SM = Sinomenine, NC = Normal Control, NS = Non-significant.

Infarct area commonly used for the estimation the myocardia disease. During the myocardial injury increase the size of infarct area. No infarct area was observed in the normal rats. I/R induced rats exhibited the boosted infarct area which was suggesting the induction of cardiac disease and sinomenine treated rats significantly (P < 0.001) suppressed the infarct area (Fig. 4) and exhibited the cardioprotective effect.

### 3.4 Platelet aggregation parameters

**Fig. 5:**
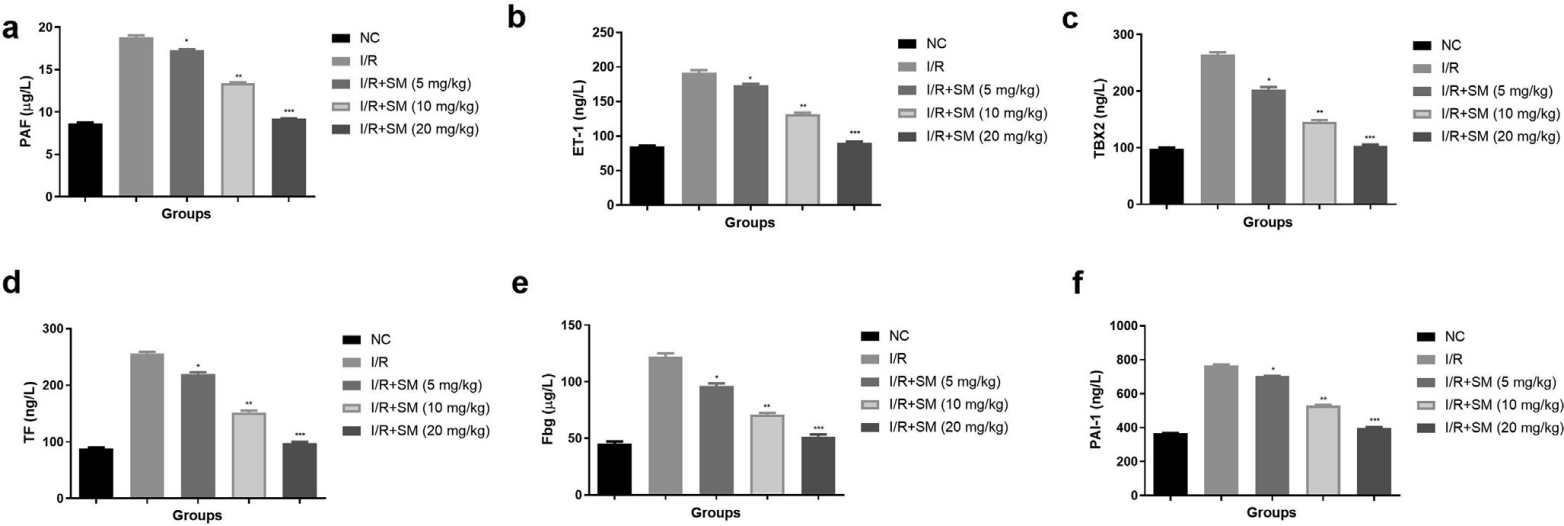
Exhibited the platelet aggregation parameters. **a:** PAF, **b:** ET-1, **c:** TBX2, **d:** TF, **e:** Fbg and **f:** PAl-1. Data were presented as mean ± SEM. Tested group rats compared I/R where *P < 0.05, **P < 0.01 and ***P < 0.001. Where I/R = Ischemia reperfusion, SM = Sinomenine, NC = Normal Control, PAF = Platelet activating factor, ET-1 = Endothelin, TBX2 = Thromboxane B2, TF = Tissue factor, Fbg = Plasma fibrinogen, PAI-1 = Plasminogen activator inhibitor 1.

Myocardial I/R induces the endothelial injury which activates the fibrinolytic system, blood clotting, platelet system and endothelium system to participate in repairing. I/R induced rats displayed the enhanced level of PAF (Fig. 5a), ET-1 (Fig. 5b), TBX2 (Fig. 5c), TF (Fig. 5d), Fbg (Fig. 5e), PAl-1 (Fig. 5f) and suggesting the myocardial injury. Sinomenine treatment significantly (P < 0.001) suppressed the level of platelet aggregation parameters and suggesting the myocardial protective effect.

### 3.5 Cardiac parameters

**Fig. 6:**
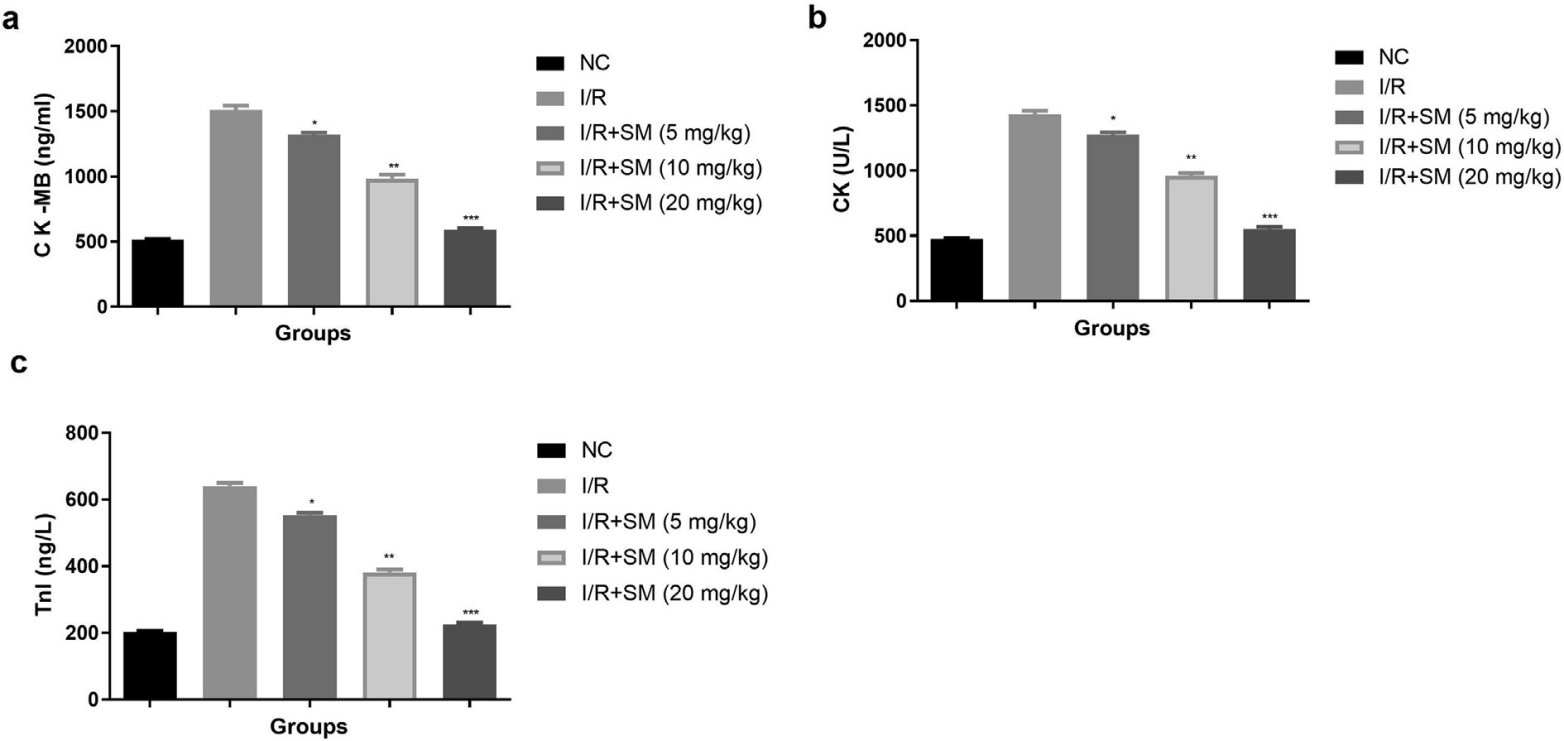
Exhibited the cardiac parameters. **a:** CK-MB, **b:** CK and **c:** Tnl-1. Data were presented as mean ± SEM. Tested group rats compared I/R where *P < 0.05, **P < 0.01 and ***P < 0.001. Where I/R = Ischemia reperfusion, SM = Sinomenine, NC = Normal Control, CK-MB = Creatine kinase MB, CK = Creatine kinase, TnI = Troponin I.

The myocardial enzymes such as Tnl, CK and CK-MB are the significant marker to use to estimation the degree of myocardial injury. During the myocardial injury, the level of myocardial parameter increased. The level of myocardial parameter within range observed in the normal group and I/R group exhibited the enhanced level of Ck-MB (Fig. 6a), Ck (Fig. 6b), Tnl (Fig. 6c) and sinomenine treatment significantly (P < 0.001) repressed the cardiac parameters.

### 3.6 Antioxidant parameters

**Fig. 7:**
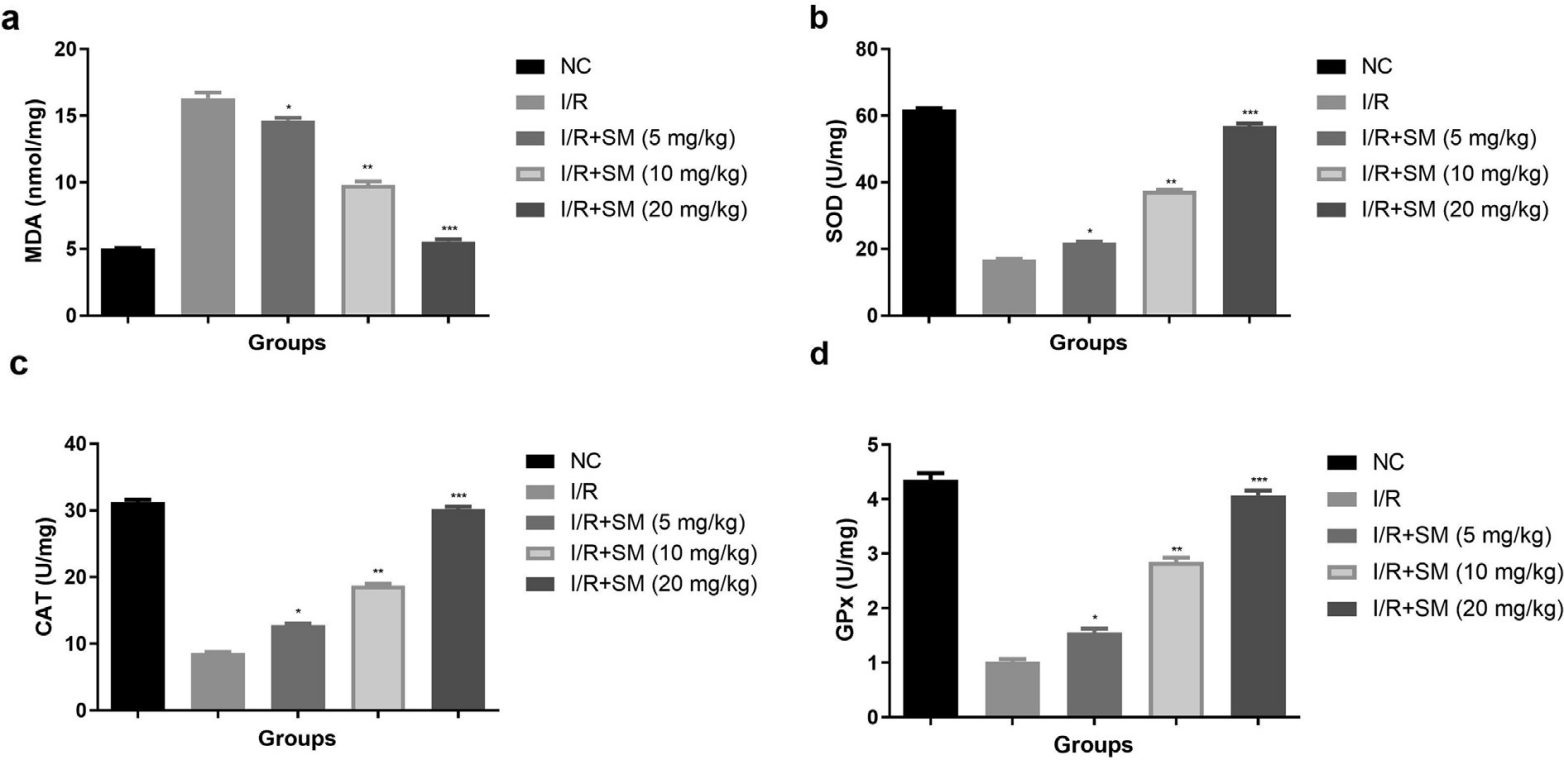
Exhibited the antioxidant parameters. **a:** SOD, **b:** CAT, **c:** MDA and **d:** GPx. Data were presented as mean ± SEM. Tested group rats compared I/R where *P < 0.05, **P < 0.01 and ***P < 0.001. Where I/R = Ischemia reperfusion, SM = Sinomenine, NC = Normal Control, MDA = Malonaldehyde, GPx = Glutathione peroxidase, CAT = Catalase, SOD = Superoxide dismutase.

SOD, CAT and GSH are the significant antioxidant enzymes and MDA exhibit the level of lipid peroxide and use for the estimation of oxidative stress. Oxidative stress is a major contributor to the progression of heart disease. It's no secret that oxidative stress exacerbated the I/R injury. In this investigation, I/R induced group rats had enhanced level of MDA (Fig. 7a) and lower levels of SOD (Fig. 7), CAT (Fig. 7c) and GPx (Fig. 7d). Sinomenine therapy considerably (P < 0.001) increased SOD, GPx, CAT, and lowered MDA levels.

### 3.7 LDH and AST

**Fig. 8:**
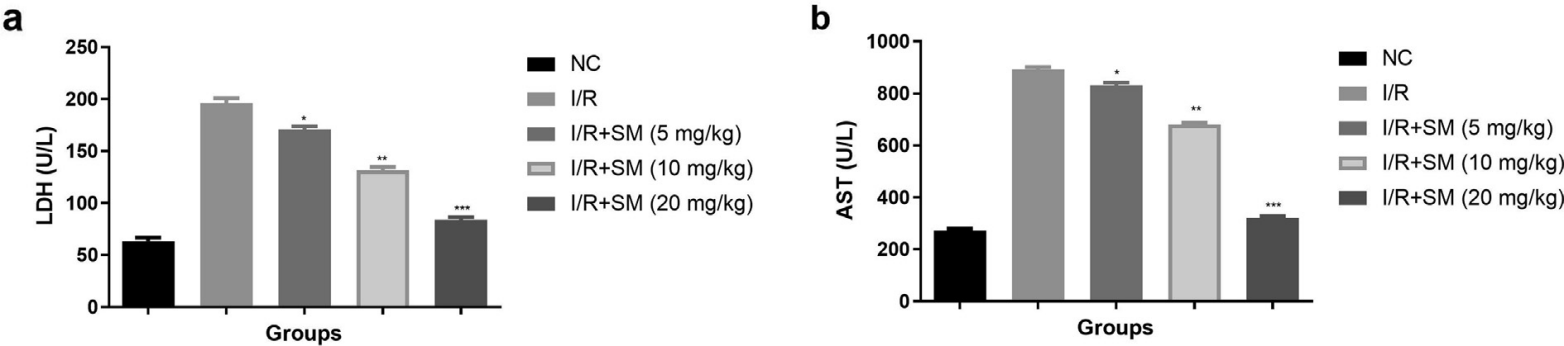
Exhibited the LDH and AST parameters. **a:** LDH and **b:** AST. Data were presented as mean ± SEM. Tested group rats compared I/R where *P < 0.05, **P < 0.01 and ***P < 0.001. Where I/R = Ischemia reperfusion, SM = Sinomenine, NC = Normal Control, AST = Aspartate aminotransferase, LDH = Lactate dehydrogenase.

LDH and AST are considered as the significant marker for myocardial injury. Both parameters exhibited the degree of myocardial injury. The level of LDH (Fig. 8a) and AST (Fig. 8b) were higher observed in I/R group and sinomenine treatment (5, 10 and 20 mg/kg) significantly (P < 0.001) suppressed the level of LDH and AST.

### 3.8 Hs-CRP and MCP-1

**Fig. 9:**
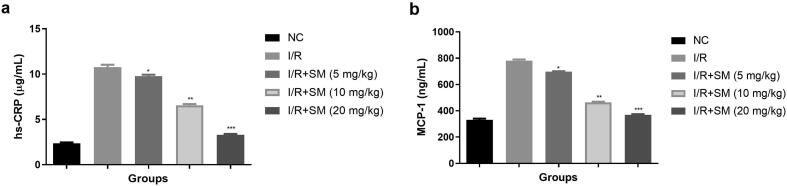
Exhibited the hs-CRP and MCP-1 parameters. **a:** hs-CRP and **b:** MCP-1. Data were presented as mean ± SEM. Tested group rats compared I/R where *P < 0.05, **P < 0.01 and ***P < 0.001. Where I/R = Ischemia reperfusion, SM = Sinomenine, NC = Normal Control, MCP-1 = Monocyte chemoattractant protein-1, hs-CRP = C-reactive protein.

MCP-1 is the monocytokines that commonly used for prediction the coronary heart disease. Hs-CRP is the marker of thrombosis, which accelerates the instability and formation of atheromatous plaques. Both the parameters used for estimation the heart vascular events. In this study, the level of hs-CRP (Fig. 9a) and MCP-1 (Fig. 9b) boosted in the I/R injury group rats and sinomenine treatment significantly (P < 0.001) suppressed the level of hs-CRP and MCP-1.

### 3.9 Inflammatory cytokines

**Fig. 10:**
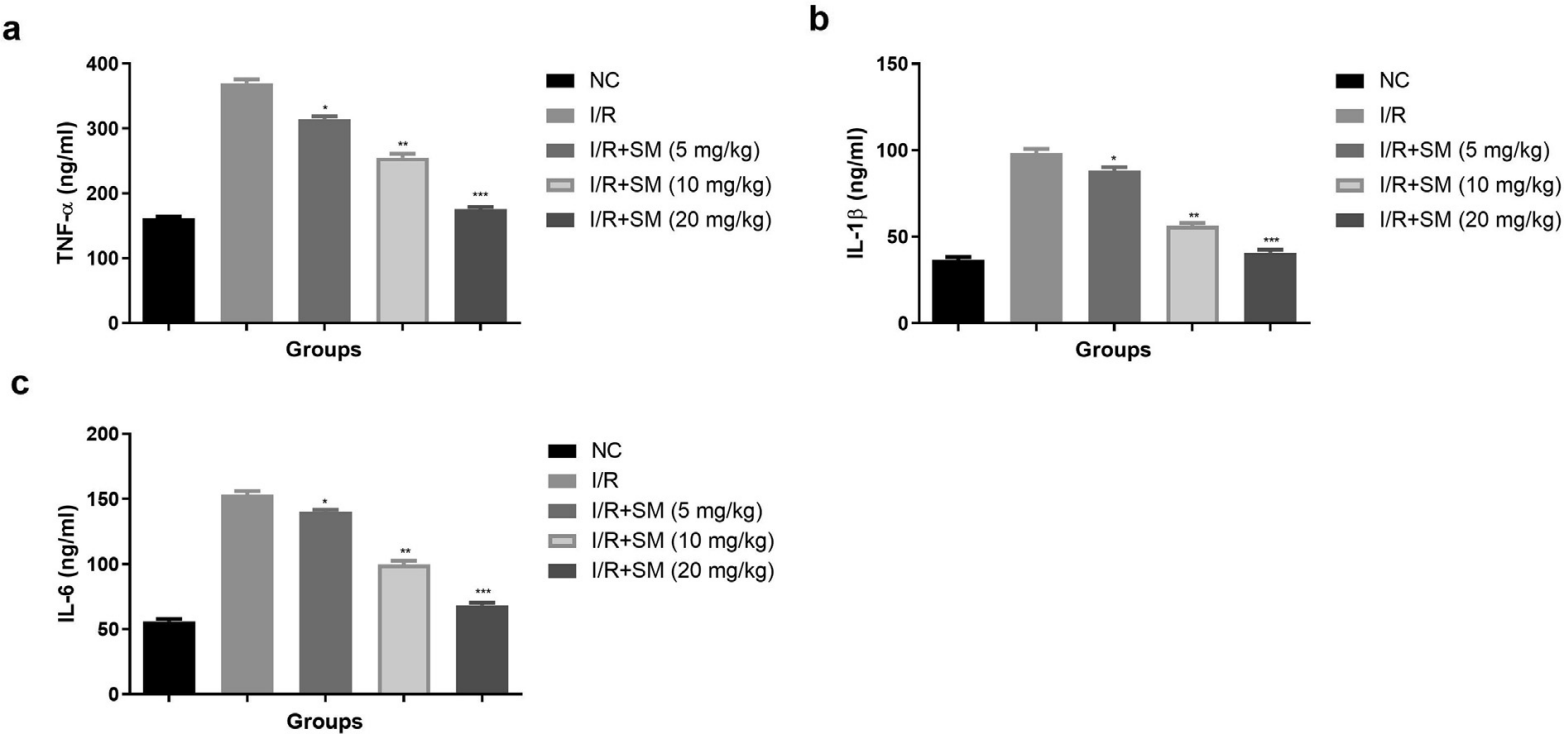
Exhibited the inflammatory parameters. **a:** TNF-α, **b:** IL-1β and **c:** IL-6. Data were presented as mean ± SEM. Tested group rats compared I/R where *P < 0.05, **P < 0.01 and ***P < 0.001. Where I/R = Ischemia reperfusion, SM = Sinomenine, NC = Normal Control, IL-1β = Interleukin-1β, IL-6 = Interleukin-6, TNF-α = Tumor necrosis factor-α.

Inflammation plays a key part in the progress of MIRI. Inflammatory factors can increase platelet adhesion, vascular endothelial damage, collagen exposure, and platelet activation. Inflammatory reaction plays a crucial role in the progression of MIRI. Inflammatory factors also boost the vascular endothelial injury, platelet adhesion, platelet activation and collagen exposure. I/R induced injury rats showed the enhanced level of TNF-α (Fig. 10a), IL-1β, (Fig. 10b), Il-6 (Fig. 10c) and sinomenine treatment significantly (P < 0.001) repressed the inflammatory cytokines.

## 4 Discussion

In this experimental protocol, we used the classical method of myocardial I/R injury to scrutinize the protective effect of sinomenine. Recently years, sinomenine has gained more popularity for improving cardiac qualities. Sinomenine suppressed inflammatory mediators, resulting in an anti-inflammatory action against STZ-induced diabetes (Li et al., 2021). Additionally, sinomenine demonstrated an anti-oxidative and hypo-lipidemic effect on the high fet diet induced atherosclerosis (Feng et al. 2019). According to this study, sinomenine may be a useful chemical for maintaining hypercholesterolemia, a key cause of cardiovascular disease, by reducing oxidative stress and improving lipid markers (Zhang et al., 2012, Yuan et al., 2018, Zhou et al., 2020). Li et al., reported the protective effect of sinomenine against isoproterenol induced myocardial infarction in experimental stays via anti-inflammatory and antioxidant effects (Li et al. 2013). In this experimental study, sinomenine exhibited an anti-arrhythmic effect in an isolated heart. Sinomenine treated group exhibited the suppression the incidence, number and duration of VF, VT and arrhythmia severity as compared to the control group. Furthermore, these findings suggested that sinomenine cardioprotective and antiarrhythmic properties may be attributable to its anti-oxidant and anti-inflammatory properties. Furthermore, the underlying mechanism of sinomenine cardioprotective action has not been extensively investigated.

Primary percutaneous coronary intervention (PCI) and systemic thrombolysis are the most commonly used for perfusion (Badalzadeh et al., 2014, Tang et al., 2020). Because it allows for the re-establishment of blood flow in the cardiac area, that has been impacted by the obstruction of a branch of the coronary artery and for the same, PCI is the most successful approach. The ischemic area is re-perfused during this process, boosting the ischemia/reperfusion event, which start the production of ROS (Han et al., 2019, Wang et al., 2020). This method increases the tissue injury (lethal reperfusion). Effective drug treatment could be used for the I/R to estimation the cardioprotective effect to protect the tissue from lethal reperfusion (Han et al., 2019, Tang et al., 2020). ROS production begins at low levels during the physiological conditions and is thought to be a significant mediator of cell apoptosis, expansion, differentiation, adhesion and senescence (Badalzadeh et al. 2014). Overproduction of oxidative stress during pathologic conditions such as I/R induces cell injury, which leads to the DNA oxidation, enhancing lipid peroxidation membrane chain reactions and changing the member fluidity (Han et al., 2019, Wang et al., 2020). Antioxidant substances are crucial in countering the damage caused by free radicals. It is widely known that during I/R injury, the antioxidant capability is suppressed, and an imbalance of oxidative/antioxidative molecules contributes to the oxidative balance in myocardial ischemia patients (Geldi et al., 2018, Li et al., 2019). The similar result was observed in the I/R group and sinomenine treated group exhibited the improved the antioxidant level and suppressed the production of free radicals.

During the expansion and pathogenesis of cardiac I/R, blood flow is blocked to activate the coagulation platelet factors and vascular endothelial cells which boots the Fbg to fibrin conversion (Han et al., 2019, Qiao et al., 2019, Tang et al., 2020). After that, the balance between the fibrinolysis system and body coagulation is obliterated and reduces the fibrinolytic activity and coagulation, which helpful for generating the thrombus on the blood vessel wall via fibrin accumulation (Najafi et al. 2018). The result showed the expansion of the acute myocardial infarction in the I/R group and sinomenine treatment considerably altered the level of platelet parameters.

Reperfusion of ischemic myocardium further aggravates tissue injury induced by ischemia despite providing cells with oxygen and trophic substances (Zhang et al., 2017, Yi et al., 2019). This injury occurs due to neutrophil infiltration from the tissue vasculature and ROS production (Najafi et al. 2018). Superoxide is a significant marker of vascular tissue I/R that begins with NADPH oxidase catalysis in neutrophils or the outflow of electron transport chain in mitochondria (Li et al., 2019, Wang et al., 2020). It is widely known that heart tissue is prone to oxidative destruction. I/R-induced oxidative stress causes the cardiac tissue injury to undergo cellular apoptosis, which can be reduced via scavenging the free radicals (Han et al., 2019, Liu et al., 2019). I/R damage is the most common cause of cardiac dysfunction, indicating that reperfusion is a key trigger for a number of processes that contribute to cardiac dysfunction caused by I/R injury (Han et al., 2019, Tang et al., 2020).

It is well documented that ROS is generated upon reperfusion of the ischemic organ rather than during ischemia (Najafi et al. 2018). The generation of ROS begins during the I/R injury, causing oxidative stress, which plays a crucial role in the I/R damage that disrupts cardiac function. The production of ROS from the reperfusion of ischemic heart during the I/R damage (Han et al., 2019, Liu et al., 2019). ROS causes DNA oxidation and membranous phospholipid protein oxidation, which are linked to the I/R pathogenesis, carcinogenesis, aging, and degenerative disease (Qu et al., 2019, Rinaldi et al., 2019). During the I/R injury, ROS starts the dysfunction in endothelial cells, cardiac myocytes and initiates the chemical reaction during the I/R injury. Ischemic cardiac tissue showed the production of ROS, during the reperfusion and could be related to the myocardial stunning, after the I/R injury reversible (Zhang et al., 2017, Li et al., 2021). During the I/R injury, start the production of ROS and starts damaging the mitochondrial DNA, that leads to more ROS generation and maybe burst production of ROS. Furthermore, myocardial stunning (dysfunction) may help to regulate the massive amount of ROS production in myocytes following an I/R injury (Najafi et al. 2018). CAT along with the SOD and GPx, play a significant role in the protection against LPO (Gatzke et al., 2018, Han et al., 2019). According to a recent study, erythrocyte reduction of CAT and SOD in acute myocardial infarction patients is caused by inactivation/alteration of these antioxidant enzymes through cross linking or exhaustion of these antioxidant enzymes through LPO (Qu et al., 2019, Li et al., 2021). During the normal process, GPx catalyses the peroxide reduction GSH utilisation as a substrate, and starts the conversion into GSSG. Other antioxidant such as GSH play a dual role as substrate in scavenging the reaction catalyzed by GPx and also scavenge the vitamin (C and E) radicals (Chen et al., 2017, Zheng et al., 2020). GSH deficit has been linked to coronary restenosis following percutaneous coronary intervention, and its deficiency has been linked to the significant postreperfusion syndrome (Liu et al., 2018, Jing et al., 2020). The reduced level of GSH may contribute to diminished the GPx activity because GSH is the one substrate of GPx. During the I/R injury, boosted the ROS production that can further detoxified the endogenous antioxidant enzymes. GPx and SOD are important enzymes that serve as free radical scavengers and may help to reduce ROS production. SOD catalyses the dismutation of the superoxide anion radical (O_2_) to H_2_O_2_, which is then scavenged to water by GPx at the expense of GSH. The findings revealed that SM has a protective effect against free radicals by increasing the levels of GPx and SOD (Chen et al., 2017, Zhang et al., 2017, Zheng et al., 2020). Sinomenine treatment considerably suppressed the MDA level and boosted the SOD, GPx, indicating the cardioprotective effect may be due to attenuating the lipid peroxidation following myocardial I/R Based on the findings, we can deduce that SM protects against I/R injury via reducing oxidative stress.

Reperfusion of the heart after an ischemic period could cause the dangerous arrhythmias. VT and VF are the most common causes of sudden death after spontaneous integrated flow restoration. According to previous study, oxygen-derived free radicals play a key role in the development of ventricular arrhythmias (Badalzadeh et al., 2014, Li et al., 2019). Sinomenine treatment considerably reduces the duration and number of VT + VF during ischemia (30 min). except this, the frequency of VF, number of VT + VF during reperfusion (120 min). The VT + VF duration after reperfusion (120 min), I/R were considerably suppressed in the myocardial infarction area, after the sinomenine treatment. The under lying reason may be the stress that might contributed to this abnormal heart rhythm causing Ventricular arrhythmias in 120 min group have a lower value than the 30 min group. Such reports are available that stress can lead Ventricular arrhythmias (Adameova et al. 2020). For estimation of the underlying mechanism of sinomenine, we determined the protective effect of sinomenine against myocardial I/R induced ventricular arrhythmias.

I/R injury leads to the induction of arrhythmias, microvascular injury, myocardial dysfunction and “no-reflow” phenomenon (Najafi et al. 2018). Previous research has suggested that necrosis, autophagy, and apoptosis are important factors in inducing cell death during the reperfusion phase of I/R injury (Geldi et al., 2018, Qiao et al., 2019). Normally, the weakness in impulse conduction or dysfunction in the impulse generation occurs due to a lack of hypoxia and ATP that results in mitochondrial dysfunction, which is considered the main parameter for inducing ischemia induced arrhythmias (Han et al., 2019, Qiao et al., 2019). But still, the main cause for induction of arrhythmias remains unexplored. But few study suggest that the ionic alteration and disturbance in the level of electrolytes across the mitochondrial and sarcolemmal, particularly enhance the concentration of Na^+^ and Ca^2+^ in the circulation (Badalzadeh et al., 2014, Li et al., 2019, Wang et al., 2020). Previous research suggests that the sarcolemmal calcium channels antagonizing showed a preventive effect against reperfusion induced arrhythmias in rats (Li et al., 2019, Wang et al., 2020). I/R induced rats exhibited an increased concentration of Na^+^ and Ca^2+^ and SM treatment considerably suppressed the concentration of Na^+^ and Ca^2+^.

The huge amounts of oxygen derived free radicals and intracellular pH alteration during the initial stage of reperfusion disprove the potential effect of reperfusion on the ischemic heart (Ito et al., 2003, Hadi and Al-Amran, 2019). The production of inflammatory cytokines and inflammatory reactions can be triggered by the excessive generation of free radicals and increased oxidative stress. Therefore, the overproduction of ROS and inflammatory reactions would be the significant pathophysiological mediators and mechanisms which are responsible for the alteration in ionic distributions and thereby reperfusion induced arrhythmias (Liu et al., 2019, Bi et al., 2020, Xin et al., 2020). The inflammatory response plays an important role in cardiac reperfusion. That increased the platelet adhesion, vascular endothelia injury, collagen exposure and platelet activation (Yi et al., 2019, Bi et al., 2020, Zhang et al., 2020). TNF- is a potent inflammatory cytokine that contributes significantly to myocardial injury. Because of the increased TNF-α level, leukocytes and endothelial cells begin to adhere and interact, and granulocyte infiltration into the I/R area increases. IL-6 and IL-1β levels are increased during I/R injury, which also increases myocardial damage by increasing endothelial cell and neutrophil adhesion (Xin et al., 2020, Zhang et al., 2020). The level of inflammatory cytokines increased after the I/R injury, and a similar result was seen in the I/R injury group rats, while SM therapy significantly reduced the level of cytokines and had an anti-inflammatory impact.

MCP-1 (monocytokines) commonly observed in the myocardial tissue and its increases monocyte/macrophage migration, which aggregates under the intima of blood vessels and suppresses their movement and chemotaxis, after becoming activated macrophages (Hadi and Al-Amran, 2019, Yi et al., 2019). During the I/R injury, boosted the hs-CRP level, which is closely related to the prognosis, severity and occurrence of atherosclerosis and acute cerebral infraction and it is considered as an important biomarker of cardiovascular disease (Yi et al., 2019, Feng et al., 2020). During the I/R injury, start the secretion of hs-CRP into the circulation which further increases the atheromatous plaques and instability (Ito et al., 2003, Hadi et al., 2013). In this study, I/R injury rats exhibited the boosted level of MCP-1 and hs-CRP and sinomenine considerably suppressed the level.

## 5 Conclusion

In short, sinomenine can suppress the myocardial infarct size along with reduction the myocardial enzyme level. The mechanism of myocardial protection of sinomenine is closely related to maintain the balance between the endogenous antioxidant enzymes and oxidation, suppress the oxidative stress along with inflammatory response, thrombosis and alter the platelet function. However, existing experimental study exhibited the exact interaction between inflammation, platelet function and oxidative stress is insufficient, and more investigation is required to fully comprehend the mechanism of sinomenine on heart protection. In future, we selected the more number of rodent to scrutinized the cardioprotective effect and explored the underlying mechanism.

## Declaration of Competing Interest

The authors declare that they have no known competing financial interests or personal relationships that could have appeared to influence the work reported in this paper.
